# High-Precision Ionosphere Monitoring Using Continuous Measurements from BDS GEO Satellites

**DOI:** 10.3390/s18030714

**Published:** 2018-02-27

**Authors:** Haiyan Yang, Xuhai Yang, Zhe Zhang, Kunjuan Zhao

**Affiliations:** 1 National Time Service Center, Chinese Academy of Sciences, Xi’an 710600, China; yyang@ntsc.ac.cn (X.Y.); zhangzhe@ntsc.ac.cn (Z.Z.); wgzhaokunjuan@126.com (K.Z.); 2 Key Laboratory of Precision Navigation and Timing Technology, Chinese Academy of Sciences, Xi’an 710600, China; 3 School of Astronomy and Space Science, University of Chinese Academy of Sciences, Beijing 100049, China

**Keywords:** BeiDou Navigation Satellite System (BDS), geostationary earth orbit (GEO), ionosphere monitoring, total electron content (TEC)

## Abstract

The current constellation of the BeiDou Navigation Satellite System (BDS) consists of five geostationary earth orbit (GEO) satellites, five inclined geosynchronous satellite orbit (IGSO) satellites, and four medium earth orbit (MEO) satellites. The advantage of using GEO satellites to monitor the ionosphereis the almost motionless ionospheric pierce point (IPP), which is analyzed in comparison with the MEO and IGSO satellites. The results from the analysis of the observations using eight tracking sites indicate that the ionospheric total electron content (TEC) sequence derived from each GEO satellite at their respective fixed IPPs is always continuous. The precision of calculated vertical TEC (VTEC) using BDS B1/B2, B1/B3, and B2/B3 dual-frequency combinationsis compared and analyzed. The VTEC_12_ precision based on the B1/B2 dual-frequency measurements using the smoothed code and the raw code combination is 0.69 and 5.54 TECU, respectively, which is slightly higher than VTEC_13_ and much higher than VTEC_23_. Furthermore, the ionospheric monitoring results of site JFNG in the northern hemisphere, and CUT0 in the southern hemisphere during the period from 1 January to 31 December 2015 are presented and discussed briefly.

## 1. Introduction

The second-generation BeiDou Navigation Satellite System (BDS), on 27 December 2012, became the third system around the world, after the Global Positioning System (GPS) and Global Navigation Satellite System (GLONASS), to provide position–navigation–time services [1,2,3,4]. It is composed of five geostationary earth orbit (GEO) satellites, five inclined geosynchronous satellite orbit (IGSO) satellites, and four medium earth orbit (MEO) satellites. Detailed information of the five GEO satellites is given in Table 1. All the BDS satellites currently transmit signals on three frequencies: 1561.098 MHz, 1207.140 MHz, and 1268.520 MHz [1]. 

Over the past two decades, the total electron content (TEC) of the ionosphere from GPS dual-frequency measurements have widely been used to study variations in the ionosphere, including its long-term characteristics, day-to-day fluctuation, and storm effects [5,6,7,8,9,10,11,12]. At a fixed position in the sky, GEO satellites, components of the BDS constellation important for ground receivers, have unique characteristics and provide more opportunities to monitor and study the various phenomena of the earth’s ionosphere. 

For ionospheric study, the TEC is computed using dual-frequency geometry free (GF) linear combination [5,6,9,12]:

(1) $$\begin{array}{cc} P_{4,km} & =P_{k}-P_{m}\\ & =\left(I_{k}-I_{m}\right)+c\left(DCB_{km}^{s}+DCB_{r,km}^{}\right)+\varepsilon_{4,km} \end{array}$$

where the subscripts *k* and *m* indicate different frequency. *P* denotes the code measurements (m). *I* is the ionospheric delay along the satellite signal propagation path, which can be written as a function of the slant TEC (STEC) (expressed in TECU, 1 TECU = 10^16^ electrons/m^2^) [12]:

(2) $$I_{k}=\frac{40.28}{f_{k}^{2}}10^{16}STEC_{k}$$

$DCB_{km}^{s}=d_{k}^{s}-d_{m}^{s}$ and $DCB_{r,km}^{}=d_{r,k}-d_{r,m}$ are differential code biases of the satellites and receivers in ns, respectively. $d_{}^{s}$ and $d_{r}$ is code hardware delay of satellite and receiver in ns, respectively. *f* is the frequency of the BDS signal in Hz. *c* is the speed of light. *ε* is the combination of the measurement noise and the multipath in the meter.

The STEC can be easily expressed as a function of TEC from Equations (1) and (2):

(3) $$STEC_{km}=\mu_{km}\left(P_{4,km}-c\cdot DCB_{km}^{s}-c\cdot DCB_{r,km}^{}+m_{4,km}+\varepsilon_{4,km}\right)$$

where $\mu_{km}=\frac{f_{k}^{2}f_{m}^{2}}{40.28\cdot 10^{16}\left(f_{m}^{2}-f_{k}^{2}\right)}$ is a coefficient used to convert TECU to length units (m).

Because of the satellite signals pass the whole ionosphere (60~1000 km), users usually assumed that all electrons in the ionosphere were concentrated in a thin layer around the earth at an altitude about 350 km. The intersection point between the line of the satellite to the receiver and the layer is called the ionospheric pierce point (IPP). 

In order to easily study the various behaviors of ionosphere, the STEC can be converted into vertical TEC (VTEC) at IPP using the modified single-layer model (MSLM) mapping function [13,14]:

(4) $$VTEC_{km}=STEC_{km}\cdot \cos\left(\arcsin\left(\frac{R_{e}}{R_{e}+H}\sin(\alpha z)\right)\right)$$

where *R_e_* is the mean radius of the Earth. *H* is the height of the single layer above the earth’s surface. *z* is the zenith distances at site. *H =* 350 km and *α =* 0.9782 are the recommended values of Bernese software [14].

As carrier phase measurements are much less affected by measurement noise and multipath than code measurements, but its ambiguity algorithm is more difficult, the carrier phase ionosphere observations are used to smooth the code ionospheric observations.

There have been a few studies on ionospheric monitoring using GEO satellites. Wu et al. (2013) investigated ionospheric TEC monitoring using BDS GEO measurements at the initial stage of BDS operation in China, and preliminarily analyzed the precision [15]. Kunitsyn et al. (2016) investigated the application of satellite-based augmentation systems (SBASs) L1/L5 (L1 = 1575.42 MHz, L5 = 1176.45 MHz) dual-frequency combination in ionospheric monitoring, and the results indicate that this method can be used to study the relative variation of TEC at a fixed IPP, but it cannot obtain its absolute values [16]. Other studies in this field have mainly focused on the ionospheric storm monitoring [11,17].

Recently, with the continuous development of BDS, there are now more than 120 International GNSS Service (IGS) sites around the world that can receive BDS signals [18]. More and more GEO measurements can be used in ionospheric monitoring applications. The purpose of this study is to make a more detailed analysis of the advantage of using BDS GEO to monitor ionosphere and provide more valuable monitoring results based on a large amount of measurements. The structure of this article is as follows: in Section 2 and Section 3, the continuity and the precision of computed VTEC using BDS GEO satellites are analyzed in detail. In Section 4, the monitoring results of CUT0 station and JFNG station in 2015 are presented and discussed briefly. Finally, a summary and conclusion are presented regarding the main advantage of GEO satellites in ionospheric monitoring.

## 2. Data Source and Continuity Analysis

In order to compute the result, we selected eight sites from the IGS tracking network. The longitude of most of the sites is between 100° E and 126° E; the KZN2 and KRGG sites are not within this range because there are no ideal sites at about 55° N and 55° S that can be used to track BDS GEO satellites. Table 2 lists the receiver types, antenna types, and approximate coordinate of all sites, which are equipped with high-performance receivers and choke-ring antennas, which ensure high quality measurements. The latitude of the sites increases from the equator to the northern and southern poles, respectively. The observation period is from 1 January 2015 to 31 December 2015, and the measurement sampling interval is 30 s. The precise coordinates of the sites and precise satellite orbit were calculated using the improved Bernese software by the National Time Service Center (NTSC). The DCB data and precision can be extracted from the MGEXyyyy.bsx files, which are provided by IGS [18]. The raw observations were preprocessed using the improved Bernese software, including gross error detection, cycle slip detection, the smoothing code, and others. However, the ionospheric TEC calculation and accuracy analysis software module BDS is compiled by Matlab and are integrated into the Bernese software. 

In order to more clearly describe the advantage of BDS GEO satellites in ionospheric monitoring, we will compare and analyze the different characteristics in satellite visibility, satellite elevation, and variation in the IPP for the GEO, IGSO, and MEO satellites. 

**Figure 1:**
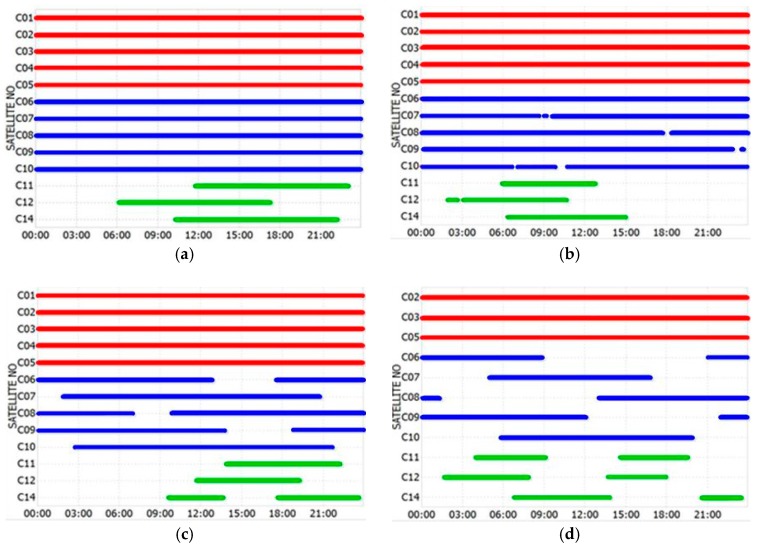
BDS satellite visibility at four sites in the northern hemisphere on Day 029/2015. SIN1 (**a**), DLTV (**b**), JFNG (**c**), and KZN2 (**d**). Red, blue, and green represent GEO, inclined geosynchronous satellite orbit (IGSO), and medium earth orbit (MEO) satellites, respectively.

Figure 1 presents the visibility of BDS satellites at four sites in the northern hemisphere on Day 029/2015. The latitudes of the four sites increase from 1.34° to 55.8° N in turn. It can be seen that there are significant differences in the observed sequence lengths of GEO, IGSO, and MEO satellites at different sites. GEO satellites C01–C05 are continuously tracked by all sites in the low latitude region, but some GEO measurement sequences with several hours of interruption occur at KZN2 site, which is mainly due to the fact that the elevation for GEO satellites is very low and close to invisible. For the IGSO satellites, the daily data gap increases continuously with the site latitude, when the site latitude is above about 10° S and 10° N. The length of the MEO measurement sequence is approximately 6–12 h/day. The analysis results of the other four sites in the southern hemisphere are similar to those of the northern hemisphere, which is not shown here. Consequently, the continuity of TEC sequences obtained from different types of satellites will be markedly different. 

**Figure 2:**
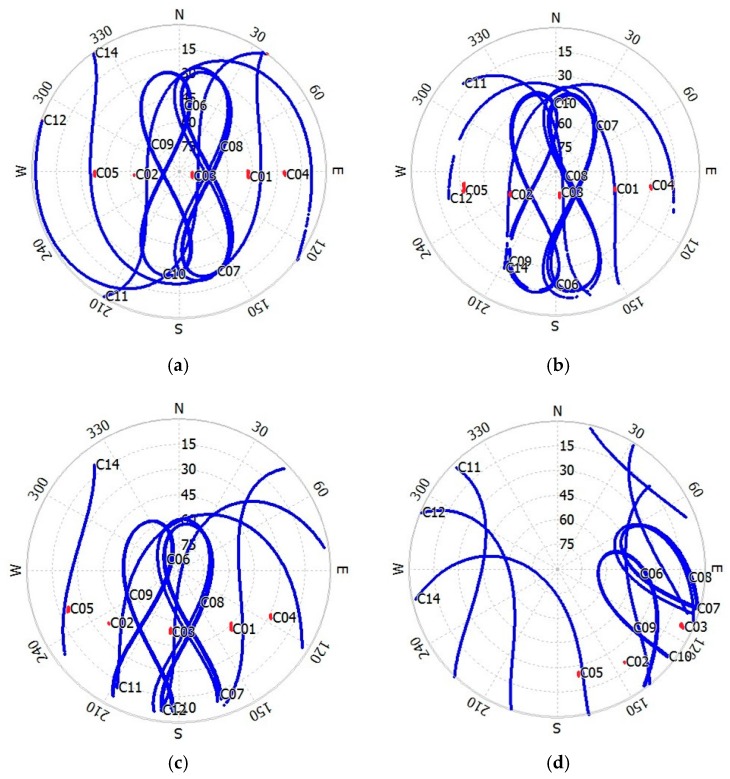
Sky plots of BDS satellites on Day 029/2015. SIN1 (**a**), DLTV (**b**), JFNG (**c**), and KZN2 (**d**). Red represents GEO satellites, and blue represents IGSO and MEO satellites.

Figure 2 presents the sky plots of all satellites against the satellite azimuths and elevations at four sites on Day 029/2015. It can be seen that the satellite elevation setting for the raw data of each site is different; however, this does not affect our analysis. These figures clearly show the elevation and azimuth variation of GEO, IGSO, and MEO at each site at different latitudes. The elevation of GEO is almost constant, but that of IGSO and MEO varies greatly. Similarly, a comprehensive consideration of the sites in the southern hemisphere shows that the satellite elevation of GEO decrease with the latitude of the site, and will become almost invisible when the latitude of the site increase above 67° (at CAS1). Therefore, it is impossible to monitor ionospheric TEC using BDS GEO satellites in the Antarctic and Arctic regions.

**Figure 3:**
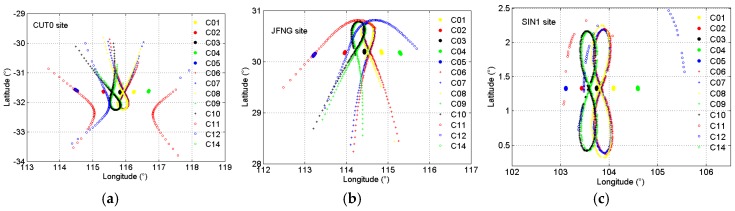
Ionospheric pierce point (IPP) of BDS satellites observed at site CUT0 (**a**), JFNG (**b**), and SIN1 (**c**) on Day 029/2015.

The GEO satellites are almost stationary compared to the large amplitude motions of the IGSO and MEO satellites, and motions of the corresponding IPPs are also negligible. The position of the IPP can be calculated by making use of the geometric relationship between the sites and satellites. Figure 3 presents the movement track of the IPP for all GEO, IGSO, and MEO satellites at sites CUT0, JFNG, and SIN1 on Day 029/2015. We can see that the movement track of the IPP for GEO, IGSO, and MEO satellites is similar to that of the satellites itself shown in Figure 2. The IPPs of five BDS GEO satellites are almost stationary, their variation range is less than 0.1° in both latitude and longitude. However, the variation range of IGSO and MEO satellites is greater than 1° in both latitude and longitude, which is much greater than that of GEO satellites. Detailed analyses indicate that the motion velocity of GEO satellites relative to ground receivers is less than 0.1 km/s in most cases, whereas that of MEO and IGSO satellites is greater than 2 km/s. Correspondingly, the IPP motion velocity of GEO satellite is less than 1 m/s, whereas that of MEO and IGSO satellites reaches several tens of meters per second. 

Based on the above analysis, the IPP of the GEO satellite compared to the MEO and IGSO satellite is almost motionless, and the high-precision TEC at the fixed IPP can be calculated continuously; the TEC sequence only changes over time and can be directly used for the study of time-dependent characteristics of the ionosphere. However, for MEO and IGSO satellites, the ionospheric TEC simultaneously varies with time and space, thus, it is impossible to calculate a continuous sequence of TEC at a fixed point directly. To study the variation characteristics of ionospheric TEC, we must adopt a method of modeling or interpolation, which can introduce a model error; and there might be an especially large error when an ionospheric anomaly is present.

## 3. Calculation Precision of TEC Using BDS GEO Observations

The STEC can be obtained from the dual-frequency GF combination using Equation (3), and the STEC can be converted into the VTEC using Equation (4). The error of the projection function is small and can be neglected when the satellite elevation is greater than 30° [15]. Following the law of error propagation, the total error $\sigma_{VTEC_{km}}$ of VTEC computation in TECU can be expressed as

(5) $$\sigma_{VTEC_{km}}=\sqrt{{\left(c\cdot \mu_{km}\cdot \sigma_{SDCB_{km}}\right)}^{2}+{\left(c\cdot \mu_{km}\cdot \sigma_{RDCB_{km}}\right)}^{2}+{\left(\mu_{km}\sigma_{P_{k}-P_{m}}\right)}^{2}}$$

where $\sigma_{SDCB}$ and $\sigma_{RDCB}$ represent the error of the DCB for satellite and receiver in ns, respectively, which can be extracted from the IGS bias products [18]. $\sigma_{P_{k}-P_{m}}=\sqrt{{\left(\sigma_{P_{k}}\right)}^{2}+{\left(\sigma_{P_{m}}\right)}^{2}}$ is the combination of multipath and measurement noise for frequency *k* and *m* in units of meter.

For BDS triple-frequency (B1, B2, and B3) measurements, three different dual-frequency GF combinations can be formed, and the VTEC calculation using the three combinations are denoted as VTEC_12_, VTEC_13_, and VTEC_23_, respectively. The corresponding coefficient is $\mu_{12}=8.9977$, $\mu_{13}=11.7579$, and $\mu_{23}=38.3902$, respectively. 

Table 3 and Table 4 present the specific values of each parameter, and the VTEC calculation accuracy using the code and carrier phase observations, respectively. The values of $\sigma_{SDCB}$ and $\sigma_{RDCB}$ in Table 3 and Table 4 are the averages during the years from 2013 to 2015. Given the other parameters in Equation (5), the code measurement noise and multipath (CMN) and carrier phase measurement noise and multipath (PMN) must be analyzed in detail. We used the code and carrier phase observations of 47 IGS stations collected from 1 January 2013 to 30 April 2015 and evaluated the CMN and PMN of the BDS GEO. The results have been published in the literature [19], and we will not conduct further analysis here.

The maximum PMN is typically a quarter of the wavelength [13], which is two orders of magnitude smaller than its influence on the code. The CMN can be estimated with a linear combination of the code and carrier phase provided by the TEQC software [20]. The average of the GEO CMN is presented in Table 3.

The carrier phase wavelength of B1, B2, and B3 is 19.20 cm, 24.83 cm, and 23.63 cm, respectively, and the maximum PMN is 4.8 cm, 6.2 cm, and 5.9 cm, respectively. In fact, we can use the triple-frequency ionosphere-free and geometry-free combinations of the carrier phase measurements to calculate the combined PMN of the three frequencies [21,22,23,24]. Assuming that the PMN at the three frequencies employed is equal and uncorrelated, the average of PMN at a single frequency is 2.64 mm [19]. Therefore, the total error of VTEC_12_ based on carrier phase observations will be less than 0.69 TECU, which is more accurate than that of the code. However, it is very difficult to handle the carrier phase ambiguity parameters, so the follow-up analysis is based on the code and smoothed code observations.

It can be clearly seen that TEC_23_ has the lowest precision because the combination coefficient $\mu_{23}$ is nearly 3~4 times larger than $\mu_{12}$ and $\mu_{13}$, and the corresponding error magnification is also larger by a factor of 3~4. 

**Figure 4:**
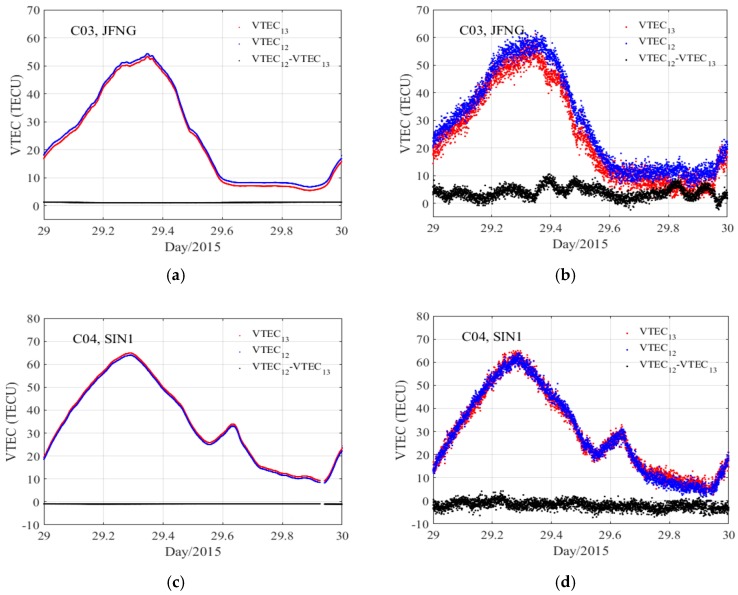
VTEC_12_, VTEC_13_, and the difference between the two groups for GEO satellites on Day 029/2015. Smoothed code: JFNG (**a**), SIN1 (**c**); Code: JFNG (**b**), SIN1 (**d**).

Figure 4 shows the difference between VTEC_12_ and VTEC_13_ for sites JFNG and SIN1 on Day 029/2015. We can see that the trend of VTEC based on the code and smoothed code is consistent, and the values are close. There is little difference between VTEC_12_ and VTEC_13_. The calculation results of eight sites in Table 1 indicate that the standard deviation (STD) of the difference between the B1/B2 and B1/B3 GF combination based on code observation is about 1.30–3.02 TECU, whereas that of the smoothed code observation is less than 0.1 TECU. This is primarily because the multipath and measurement noise of the code GF combination is far greater than that of the smoothed code GF combination. According to the previous analysis, we find that the VTEC_12_ has the highest precision, so only the combination B1/B2 will be used in follow-up analysis.

**Figure 5:**
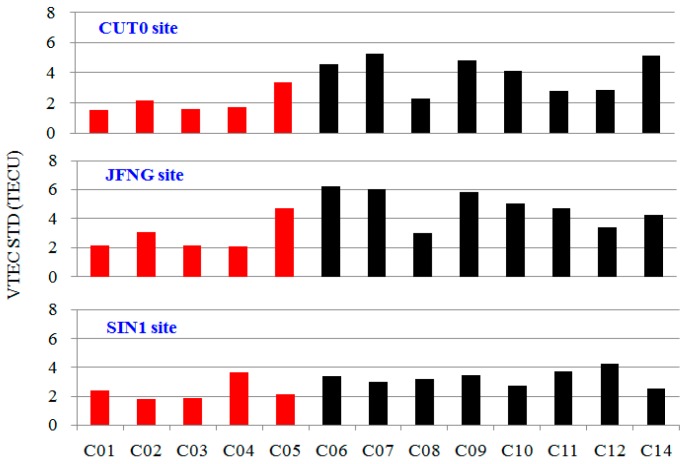
STD of differences between IGS GIM and VTEC_12_ for BDS satellites at site CUT0, JFNG, and SIN1 from 1 January 2015 to 31 December 2015. Red represents GEO satellites, and black represents IGSO and MEO satellites.

The IGS Working Group on Ionosphere provides global ionosphere map (GIM) products, which are estimated using GPS and GLONASS measurements from global sites. The final IGS ionosphere products have a temporal resolution of 2 h and a spatial resolution of 5 × 2.5° (longitude × latitude), and the GIM precision is 2–8 TECU [24]. Figure 5 presents the VTEC STD of the difference between VTEC_12_ derived from the smoothed code and IGS GIM interpolation at the same IPP at site CUT0, JFNG, and SIN1 from 1 January 2015 to 31 December 2015. We can see that the VTEC STD of GEO is generally smaller than that of MEO and IGSO. We also find, by comparing the five GEO satellites, that the STD value is related to the satellite elevation to a certain extent, and the satellites with lower elevation have a larger STD. This is related to the uniformity of IGS GIM precision, multipath and measurement noise, and others. It should be noted that the precision of the GIM is lower than that of VTEC_12_, and it is only proved that the calculation results are correct, but the precision of VTEC_12_ cannot be assessed.

## 4. Discussion

In this section, we select three sites named CUT0, SIN1, and JFNG for analysis, as they are located in the southern hemisphere, near the equator, and in the northern hemisphere, respectively. The VTECs are computed using the smoothed code observations during 2013 and 2015. Some analysis results of the ionospheric TEC sequence variation over time are used to demonstrate the characteristics of the GEO satellites in ionospheric monitoring.

**Figure 6:**
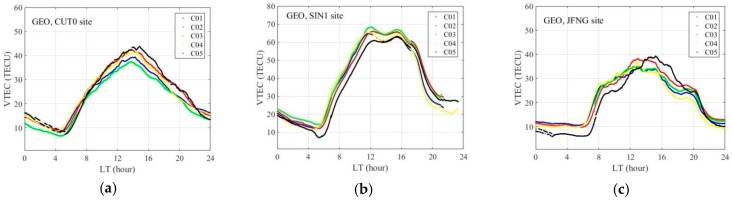
Diurnal variation of VTEC derived from five BDS GEO satellites on Day 004/2014. CUT0 (**a**), SIN1 (**b**), JFNG (**c**).

Figure 6 presents the continuous VTEC derived from five GEO satellites at CUT0, JFNG, and SIN1 site on Day 004/2014. We can see that there are slight differences in the absolute VTEC value for different satellites at the same site, primarily because the IPP for each satellite is not the same. The VTEC variations clearly show two extremum points, the peak and the minimum, which are closely related to the local solar altitude. It can be seen that for the sites CUT0 and SIN1, the minimum appears at about 5:00, but for the site JFNG it consistently lasts until about 6:30. The difference between the extremum points is clearer than that of the minimum points. The maximum values of the VTEC for site CUT0 appear at approximately 14:00, those for site SIN1 appear at 12:00, and some increasing values of VTEC can be also seen at about 15:00, and for site JFNG the maximum appears for different satellites at different times. The time of the maximum VTEC and the VTEC sequence for C05 at sites CUT0 and JFNG are significantly different from that of other satellites, and this may be related to projection function error and the quality of the observation after the initial analysis. The measurement noise and multipath of C05 is larger than that of other satellites, because the satellite elevation of C05 is very low, about 17°.

**Figure 7:**
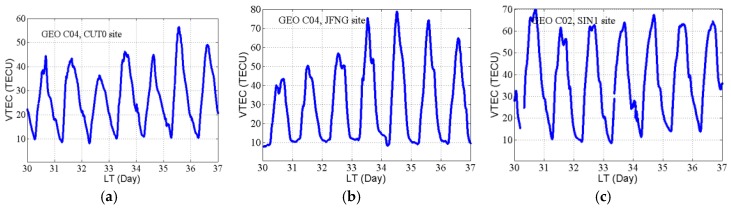
Day-to-day variation of VTEC derived from GEO satellites on Days 30–36/2014. CUT0 (**a**), JFNG (**b**), SIN1 (**c**).

Figure 4 and Figure 6 show the continuity of the ionospheric TEC within a day, and the continuity between days cannot be seen. Figure 7 displays the continuous VTEC derived from BDS GEO satellites at site CUT0, JFNG, and SIN1 during Days 030–036/2014. We also see that the VTEC varies periodically over the day and has almost the same minimum daily values of about 10 TECU, but there is a significant difference between the maximum daily values. This is related to the daily solar radiation intensity, geomagnetic intensity, and many other factors. 

**Figure 8:**
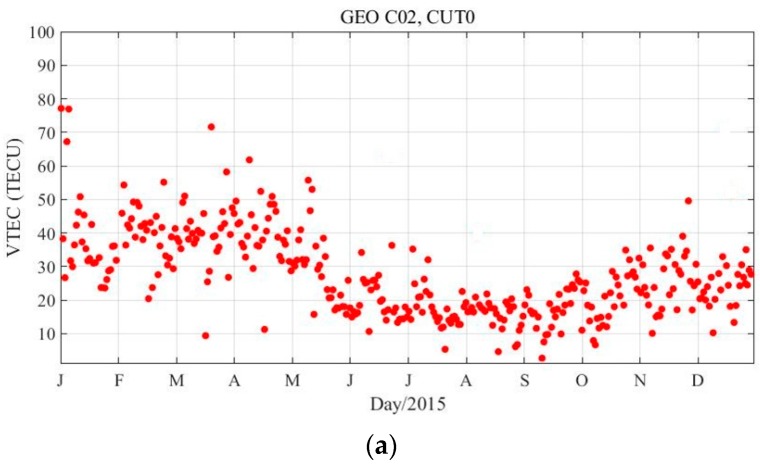

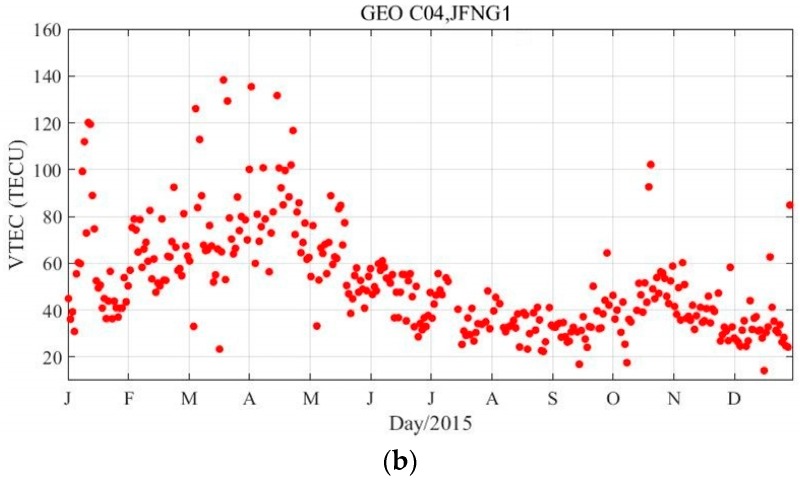
Daily maximum VTEC variation for GEO satellites in 2015. CUT0 (**a**), JFNG1 (**b**).

The daily maximum VTEC can also reflect the ionization level and is usually used to study the long-term variation of ionosphere and ionospheric anomalies. Figure 8 presents the daily maximum VTEC derived from GEO at sites CUT0 and JFNG in 2015. There are some gaps in Figure 8, which are due to no measurements on the corresponding day. Daily maximum VTEC seasonal variations can be observed, where two peaks appear in the spring and autumn, respectively. This is consistent with ionospheric annual variation [6,9,11]. Clearly, there are many ionospheric abnormal spots in the sequence shown in Figure 8. Several studies [10,15,16,17,25,26,27,28] demonstrate that the ionospheric anomaly is related to several factors such as solar activity (sunspot number), geomagnetic activity, and earthquakes. We researched a large amount of information and found the following:

○ The sunspot number increases at the beginning of January and April and at the end of March 2015.
○ The geomagnetic activity around 17 March 2015 is very strong.
○ There is moderate intensity geomagnetic activity at the end of December 2015.
○ Magnitude-8.2 and 7.7 earthquakes occurred on 21 October and 25 November 2015 in Vanuatu and Peru, respectively.

The above times match the anomalous times of the ionosphere in Figure 8, and this can, to a certain extent, reasonably explain the abnormal ionosphere phenomenon. Therefore, the TEC at the fixed IPPs based on the GEO satellites can truly present the detailed ionospheric variation characteristics, including the ionospheric variation law over time and anomaly monitoring.

## 5. Summary and Conclusions

This article focuses on the advantages of using GEO satellites to monitor the ionospheric TEC. The main advantage of this method is that the IPP of GEO is almost motionless compared to that of IGSO and MEO. A large amount of observations from eight IGS sites selected from the IGS tracking network were used for targeted analysis.

The observations of GEO are always continuous in comparison with those of IGSO and MEO satellites. The IPP motion velocity of the GEO satellite is less than 1 m/s, whereas that of MEO and IGSO satellites reaches several tens of meters per second. Combining the analysis of the site coordinates, it was shown that it is impossible to monitor the ionospheric TEC using the BDS GEO satellites in the Antarctic and Arctic regions, as the GEO satellites here are invisible or close to the horizon.

A theoretical precision analysis of the method based on the actual observations was performed. It was shown that B1/B2 is the best performance combination, and the precision of VTEC derived from the smoothed code and the raw code is 0.69 TECU and 5.54 TECU, respectively, both of which are, respectively, slightly higher than that of the combination B1/B3 and much higher than that of the combination B2/B3. 

From the perspective of application, the monitoring results of the CUT0 and JFNG site in 2015 were presented and discussed briefly. The calculated TEC at the fixed IPP of the GEO satellites demonstrates the detailed ionospheric variation characteristics, including the ionospheric variation over time and anomaly monitoring.

As the number of available GEO (SBAS system) and tracking sites increase, dual-frequency measurements will increasingly be used for ionospheric monitoring, especially during ionospheric storms, scintillations, and other abnormal phenomena. Research and analysis in this area will continue in the future.

## Figures and Tables

**Table 1:** Information on BeiDou Navigation Satellite System (BDS) geostationary earth orbit (GEO) satellites [1].

PRN	Satellite Name	Longitude Degree	Launch Date
C01	BDS-G1	140° E	16/01/2010
C02	BDS-G6	80° E	25/10/2012
C03	BDS-G3	110.5° E	02/06/2010
C04	BDS-G4	160° E	31/10/2010
C05	BDS-G5	58.75° E	24/02/2012

**Table 2:** Information about all sites.

No.	Site	Coordinates	Receiver Type	Antenna Type
1	KZN2	55.80° N, 49.12° E	TRIMBLE NETR9	TRM59800.00
2	JFNG	30.52° N, 114.50° E	TRIMBLE NETR9	TRM59800.00
3	DLTV	11.95° N, 108.48° E	TRIMBLE NETR9	JAVRINGANT_DM
4	SIN1	1.34° N, 103.68° E	TRIMBLE NETR9	LEIAR25.R3
5	XMIS	10.45° S, 105.69° E	TRIMBLE NETR9	JAVRINGANT_DM
6	CUT0	32.00° S, 115.90° E	TRIMBLE NETR9	TRM59800.00
7	KRGG	49.35° S, 70.26° E	LEICA GR10	LEIAR25.R4
8	CAS1	66.28° S, 110.52° E	TRIMBLE NETR9	TRM59800.00

**Table 3:** Error of vertical total electron content (VTEC) derived from the code observation [19].

Signal	$\mu_{km}$	$\sigma_{SDCB_{km}}$(ns)	$\sigma_{RDCB_{km}}$(ns)	$\sigma_{P_{k}}$(m)	$\sigma_{P_{m}}$(m)	$\sigma_{VTEC_{km}}$(TECU)
B1/B2	8.9977	0.13	0.21	0.47	0.39	5.54
B1/B3	11.7579	0.17	0.22	0.47	0.38	7.17
B2/B3	38.3902	0.08	0.11	0.39	0.38	20.91

**Table 4:** Maximum error of VTEC derived from the carrier phase observation.

Signal	$\mu_{km}$	$\sigma_{SDCB_{km}}$(ns)	$\sigma_{RDCB_{km}}$(ns)	$\sigma_{P_{k}}$(m)	$\sigma_{P_{m}}$(m)	$\sigma_{VTEC_{km}}$(TECU)
B1/B2	8.9977	0.13	0.21	0.048	0.062	0.90
B1/B3	11.7579	0.17	0.22	0.048	0.059	1.42
B2/B3	38.3902	0.08	0.11	0.062	0.059	3.56
